# Maternal Low‐Fat and High‐Fat Diet Decreases Survival and Alters Cytokine Signaling in Neonatal Mice With *Staphylococcus epidermidis* Sepsis

**DOI:** 10.1096/fj.202502656RR

**Published:** 2026-04-13

**Authors:** Lauren Bodilly, Sarah Weiner, Kara Misel‐Wuchter, Jennifer Bermick

**Affiliations:** ^1^ Department of Pediatrics University of Iowa Iowa City Iowa USA

**Keywords:** cytokines, malnutrition, metabolome, mice, neonatal sepsis

## Abstract

Maternal malnutrition increases susceptibility to sepsis and mortality in neonates. The reason for this increased susceptibility remains unknown. We aimed to evaluate bacterial burden and serum cytokine levels in septic neonatal mice born to dams fed diets with different dietary fat content. 6‐week‐old C57BL/6 dams were placed on a low‐fat (LFD) (10% kcal from fat), control (CD) (18% kcal from fat), or high‐fat (HFD) (60% kcal from fat) diet for 3 weeks before breeding. Sepsis was induced in P4–P6 offspring via intraperitoneal *Staphylococcus epidermidis* injection. Mice were monitored for survival. At 12 h after sepsis, serum and peritoneal wash fluid were collected for bacterial count and serum cytokine levels. In the absence of infection, P4–P6 offspring had untargeted serum metabolomics performed. Septic offspring of dams fed LFD and HFD had significantly higher mortality than offspring of dams fed CD. There was no difference in serum or peritoneal wash bacterial loads. Maternal diet and *S. epidermidis* sepsis caused changes in basal serum cytokine levels, with HFD causing decreased cytokine elevation during sepsis. Maternal LFD and HFD altered similar metabolomic pathways in offspring. Maternal LFD and HFD decrease survival during neonatal sepsis and alter serum cytokines and the metabolome, supporting a role for maternal nutrition in neonatal immune function and infection susceptibility.

## Graphical abstract

Maternal dietary fat content modulates the serum metabolome and sepsis outcomes in neonatal mice. Sphingolipid metabolites, sphingosine and ethanolamine phosphate, were altered in offspring of dams fed a low‐fat (10% kcal fat) or high‐fat (60% kcal fat) diet compared to offspring of dams fed a control‐fat diet (18% kcal fat). Offspring of low‐fat and high‐fat diet fed dams had significantly higher mortality from *Staphylococcus epidermidis* sepsis. Maternal diet‐induced metabolic changes in offspring may affect sepsis survival and merit future investigation.

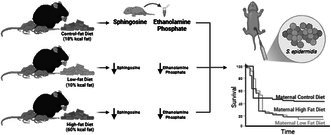

## 1 Introduction

Sepsis is a leading cause of morbidity and mortality in neonates [1]. Sepsis is a syndrome in which a life‐threatening infection leads to a dysregulated host immune response [2]. Neonatal sepsis can be early‐onset, within the first 72 h of life, or late‐onset, occurring after 72 h of life. Coagulase‐negative staphylococcus, including *Staphylococcus epidermidis* , is a common pathogen causing late‐onset sepsis. Neonatal risk factors for sepsis include, but are not limited to, prematurity, low birth weight, and meconium‐stained amniotic fluid [3]. Hospital‐associated risk factors include the presence of a central venous line and mechanical ventilation. Additionally, there are maternal risk factors such as premature rupture of membranes, infection, and maternal weight [3, 4, 5, 6]. Intrapartum antibiotic administration to pregnant individuals who are Group B *Streptococcus* (GBS) positive has significantly reduced neonatal GBS infection; however, early and prolonged antibiotic exposure may increase the risk of late‐onset neonatal sepsis, especially in premature infants [7, 8].

Maternal malnutrition is a global epidemic that can be categorized as undernutrition, overnutrition, or micronutrient deficiency or excess. Currently, 9.1% of women suffer from undernutrition and 32.5% of women suffer from overnutrition, with rates of overnutrition continuing to rise globally [9]. The following prepregnancy body mass index (BMI) is used to categorize nutritional status: BMI kg/m^2^ < 18.5 (underweight‐undernutrition), 18.5–24.9 (normal weight), 25.0–29.9 (overweight‐overnutrition), and ≥ 30 (obese‐overnutrition). Prepregnancy BMI varies across countries with underweight BMI estimated around 5% in the United States, 3% in Europe, and 17% in Asia [10]. Alternatively, pre‐pregnant overweight and obesity BMI is estimated around 42% in the United States, 30% in Europe, and 10% in Asia. The prepregnancy epidemiologic data in Africa is country dependent, with Tanzania having 6.6% underweight and 31.3% overweight/obese compared to Jordan in which 40% of women had a prepregnancy underweight BMI and 10.1% an overweight/obese BMI [11, 12]. Neonates born to mothers with undernutrition or overnutrition have increased rates of pre‐term birth, growth restriction or excess, sepsis, and mortality [4, 13, 14]. However, there remains a significant knowledge gap in understanding why maternal malnutrition increases the risk of sepsis in neonates.

The fetal programming hypothesis suggests that alterations in fetal nutrition, related to maternal nutrition and other exogenous factors, determine an individual's long‐term immunologic and metabolic health [15]. There is evidence that umbilical cord blood cell lineages are different in infants born small‐for‐gestational age compared to those born appropriate‐for‐gestational age; however, data was not collected on maternal nutrition or weight [16]. A murine maternal undernutrition model demonstrated that offspring CD4+ T cells skewed toward the TH2 lineage and resulted in an asthma‐like phenotype, highlighting the immune effects of maternal undernutrition [17]. Another murine model of maternal malnutrition utilizing a low‐protein/low‐fat diet (LFD)‐induced environmental enteropathy in neonatal offspring, characterized by an increase in intestinal neutrophils, increased inflammation, and alteration of the gut microbiome [18].

Several studies on maternal overnutrition demonstrate altered placental immunity [19, 20, 21]. Additionally, umbilical cord blood monocytes and myeloid dendritic cells from obese pregnancies have decreased lipopolysaccharide (LPS)‐induced responses [22, 23]. Dampened monocyte responses were also shown following *Escherichia coli* stimulation and were replicated in fetal peripheral monocytes and tissue‐resident macrophages in a rhesus macaque model of maternal obesity [24]. In a murine model of maternal overnutrition, offspring born to dams on obesogenic diets had increased susceptibility to bacterial infection, although this infectious challenge occurred after weaning rather than during the neonatal period [25].

Given the paucity of information on the impact of maternal malnutrition on neonatal immunity, we implemented a murine model of maternal malnutrition by exposing dams to LFD, normal‐fat diet, or high‐fat diet (HFD) and infected their offspring with *S. epidermidis* to induce late‐onset sepsis. We hypothesized that neonates born to LFD‐ and HFD‐fed dams would have dampened immune responses and decreased survival during *S. epidermidis* sepsis.

## 2 Methods

### 2.1 Animals

The investigations were conducted in accordance with the ARRIVE guidelines and were approved by the Institutional Animal Care and Usage Committees at the University of Iowa [26]. Mice were housed in a University of Iowa vivarium. Food and water were provided ad libitum. Female and male C57BL/6 mice aged 6 weeks were obtained from The Jackson Laboratories (Bar Harbor, ME, USA) or were offspring of in‐house breeding pairs of C57BL/6 dams and sires. Neonatal male and female mice born in‐house were used for all experiments.

### 2.2 Model of Maternal Malnutrition

Six‐week‐old C57BL/6 female mice were selected by cage to be placed on a LFD (Research Diets Inc. D12450J; 10% kcal provided by fat), on control‐fat diet (CD; Research Diets Inc. D17030603; 18% kcal provided by fat), or HFD (Research Diets Inc. D12492; 60% kcal provided by fat) (Table 1). The CD is a purified diet that is matched in kcal provided by fat to the grain control chow, 7913 NIH‐31 Irradiated Modified Open Formula Mouse Diet, provided by our Office of Animal Resource and matched in protein and micronutrients to the low and HFDs. Body weights were monitored weekly for 3 weeks, and the females were then mated with C57BL/6 males. After breeding, females were independently housed and assessed for pregnancy by intravaginal plug formation and visual inspection. Female mice remained on their designated diet for the entire experiment. Male mice were exposed to the LFD, CD, or HFD during the week of mating. No blinding occurred during the study as the diets are different colors and easy to distinguish.

**TABLE 1:** Diet composition of D12450J (LFD), D17030603 (CD), and D12492 (HFD) from Research Diets Inc.

Diet	D12450J	D17030603	D12492
Protein (%kcal)	20	20	20
Fat (%kcal)	10	18	60
Carbohydrate (%kcal)	70	62	20
kcal/g	3.85	4.0	5.2
*Ingredients* (g)			
Casein, 30 Mesh	200	200	200
DL‐Methionine	3	3	3
Corn Starch	506.2	427.5	0
Maltodextrin 10	125	125	125
Sucrose	68.8	68.8	68.8
Cellulose, BW200	50	50	50
Soybean Oil	25	25	25
Lard	20	55	245
Mineral Mix S100026	10	10	10
Calcium carbonate	13	13	13
Sodium chloride	5.5	5.5	5.5
Potassium citrate, 1 H_2_O	16.5	16.5	16.5
Vitamin Mix V10001	10	10	10
Choline bitartrate	2	2	2

*Note:* kcal is kilocalorie.

### 2.3 Body Composition

Total body, fat, lean, and fluid mass were quantified by NMR spectroscopy using a Bruker mini‐spec LF 90II instrument (Bruker Corporation, Billerica, MA). To analyze body composition, mice were placed into a restraint tube and inserted into the NMR machine.

### 2.4 *S. epidermidis*

*S. epidermidis* (Schroeter) Migula (ATCC 12228) was cultured at 37°C overnight in nutrient broth as seed culture to inoculate a fresh broth that was grown for 2 h. Spectrophotometry was used to estimate bacterial concentration of broth, and a growth curve was used to determine amount of broth needed to be pelleted to achieve approximately 2 × 10^9^ cfu/mL. Pellet was suspended in 1 mL of PBS (no Ca^2+^/no Mg^2+^) to produce inocula. Inocula were plated in 1:10 dilutions and incubated for 24 h. Serial dilutions resulted in a final density of 2 × 10^7^–1 × 10^9^ cfu/mL which corresponded to a dose of 1 × 10^7^–5 × 10^8^ cfu/injection.

### 2.5 Neonatal Sepsis

Neonatal mice were chosen to be infected or not infected based on litter due to the need to remain with their mothers. P4–P6 neonatal mice were weighed. Mice less than 2.25 g were excluded from the study. P4–P6 neonatal mice infected with *S. epidermidis* solution via intraperitoneal (i.p.) injection (50 μL/mouse). They remained with their mother and littermates following infection.

### 2.6 Survival Studies

After injection of *S. epidermidis* , mice were monitored for survival for 5 days. Mice were monitored every 6 h for the first 24 h and then every 12 h thereafter. An injury severity scale was used to assess the severity of symptoms. Mice were graded on a scale of normal, decreased, or absent for presence of milk spot, vigor, and if located near littermates. If their scores were absent or significantly decreased across all three categories, then they were compassionately euthanized.

### 2.7 Blood and Tissue Harvest

Neonates were euthanized at 12 h postinfection for blood and tissue harvest. Neonates were decapitated and whole blood was collected with 50 μL of heparin in a 24‐well plate. Whole blood samples were centrifuged at 3000*g* for 15 min at room temperature. The serum was aliquoted into separate microcentrifuge tubes and samples were frozen at −80°C until analysis. The intestine was exposed, and the colon was excised following identification of the cecum. The colon was stored at −20°C until analysis.

Dams were given a sublethal dose of sodium pentobarbital (150 mg/kg i.p.) and intracardiac puncture was performed to obtain whole blood once the mouse was sufficiently anesthetized. As described above, whole blood samples were centrifuged, and the serum was frozen at −80°C. Dams were cervically dislocated after intracardiac puncture.

### 2.8 Bacterial Count

Following decapitation, whole blood was collected as described above. To collect peritoneal fluid, 150 μL of sterile PBS was injected into the peritoneal cavity of neonatal mice and removed twice per animal. All samples were serially diluted in sterile PBS. The following dilutions were plated on nutrient agar plates: 1:10 and 1:100 and peritoneal fluid 1:10 through 1:1 000 000. Colony‐forming units were counted after 24 h.

### 2.9 Cytokines

Cytokine levels were quantified in neonatal serum samples using bead‐based multiplexed technology. Protein levels of the cytokines G‐CSF, GM‐CSF, TNF‐α, IL‐1β, IL‐6, IL‐10, MIP‐1α, MIP‐1β, MCP‐1, and RANTES were quantified using a 23‐cytokine multiplexed Bio‐Plex assay (#M60009RDPD) on a Bio‐Plex 200 machine following the manufacturer's instructions (Bio‐Rad, Hercules, CA, USA). Data are not shown for the rest of the panel.

### 2.10 Metabolomic Profiling

All metabolite profiling was performed by Metabolon Inc. (Durham, NC). All serum was collected from P4 to P6 mice and stored at −80 until sample processing. Briefly, serum was thawed and pooled from three to six mice per dietary intervention group to achieve 150 μL/sample. Samples were sent to Metabolon Inc. for nontargeted metabolic profiling using UHPLC–MS/MS. A total of 1034 different metabolites were identified by automated comparison of the serum samples to an in‐house library of chemical standards.

### 2.11 Metabolic Pathway Analysis

Metabolomic data was analyzed using Metaboanalyst v6.0. To perform statistical analysis (one factor) the original data file from Metabolon was saved and edited to include only offspring of LFD‐fed and grain CD‐fed dams or HFD‐fed and grain CD‐fed dams. Data was median‐centered, log‐transformed, and pareto‐scaled. Principal Component Analysis was performed with statistical significance evaluated using PERMANOVA and distributions computed using the Euclidean distance from the center. Metabolic pathway analysis was conducted to better understand the functional impact of maternal diet on neonatal metabolism. This feature combines pathway enrichment analysis and pathway topology analysis as previously described [27]. This was performed using the same data files and data preprocessing strategies as described above. The analysis was performed using the following parameters: (1) enrichment analysis was performed using the global test, (2) centrality was measured using Relative Betweenness, and (3) *Mus musculus* (house mouse) and the Kyoto Encyclopedia of Genes and Genomes (KEGG) database were used as a reference for metabolic pathways.

### 2.12 Colonic Microbiome DNA Extraction, 16S rRNA Sequencing, and Analysis

Excised colons were stored at −20°C until analysis. To ensure adequate microbial recovery, the entire colon was processed rather than isolating luminal fecal material alone. Samples were bead‐beaten in 2 mL BashingBead tubes using the BioSpec Mini‐BeadBeater‐96 (BioSpec Products, Bartlesville, OK, USA) at 2400 rpm for 5 min, followed by a 5‐min rest, for a total of four cycles. Microbial DNA was extracted using the ZymoBIOMICS DNA Miniprep Kit (Irvine, CA, USA, Cat#: D4300), following the manufacturer's protocol. Two step PCR amplification of the V3–V4 hypervariable region of the bacterial 16S rRNA gene was performed as previously described [28]. The first PCR (PCR1) amplified the V3–V4 region using high‐fidelity polymerase and region‐specific primers. A second PCR (PCR2) added sample‐specific indexed adapters to enable multiplexed sequencing. Amplicons were verified by gel electrophoresis, pooled, cleaned using magnetic beads, and quantified. PCR amplicon quality was assessed using an Agilent Bioanalyzer. Sequencing was performed on the Element AVITI24 platform at the University of Iowa Genomics Core using paired‐end reads.

Post‐sequencing, raw FASTQ data files were processed using the dada2 v1.34.0 pipeline in Rstudio v 2024.04.2 + 764 as previously described [28]. To generate amplicon sequence variants, paired reads were merged and chimeric sequences were removed. Taxonomy was assigned using the Silva v138.2 reference database. Phyloseq v1.50.0 was used to evaluate measures of α‐ and β‐diversity. Figures were created with ggplot2 v4.0.1. Depending on data normality, α‐diversity measures were compared using either the Kruskal–Wallis test or one‐way ANOVA. β‐diversity comparisons were conducted using PERMANOVA (adonis2) in the vegan v2.7‐2 R package.

### 2.13 Statistical Analysis

Data were analyzed using GraphPad Prism. For data comparison among only two groups, statistical analysis was performed using *t*‐test for parametric data and Mann–Whitney *U* for nonparametric data. For data comparison among three or more groups, statistical analysis was performed using two‐way ANOVA with the Holm–Sidak multiple comparisons. If data were not normally distributed, they were log‐transformed and analyzed for normality prior to analysis. Data are expressed as mean ± SD for parametric data and median (IQR) for nonparametric data in the text and figures. Survival analysis was performed by log rank test. A value of *p* ≤ 0.05 was considered significant.

## 3 Results

### 3.1 Maternal Dietary Fat Content Alters Maternal and/or Neonatal Weight and Body Composition

**FIGURE 1:**
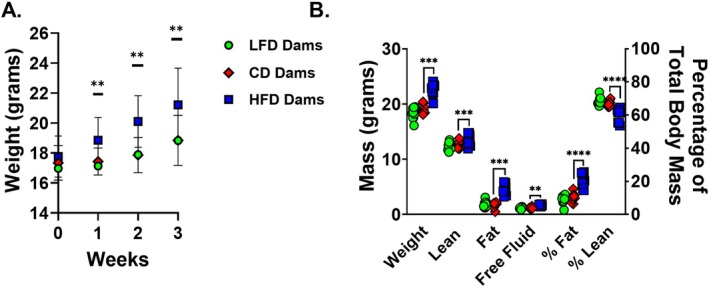
Maternal weight and body composition is impacted by high‐fat diet but not low‐fat diet. Six‐week‐old C57BL/6 dams were placed on a low‐fat diet (LFD) (10% kcal from fat), control diet (CD) (18% kcal from fat), or high‐fat diet (HFD) (60% kcal from fat) for 3 weeks before breeding. (A) LFD‐fed dams weigh the same as CD‐fed dams. HFD‐fed dams weighed more than CD‐fed dams from Week 1 through Week 3 (*n* = 15/group). Data presented as mean with SD bars. (B) After 3 weeks of dietary intervention, separate cohorts of dams underwent NMR to assess body composition (*n* = 10/group). There was no difference in body composition in LFD‐ and CD‐fed dams. HFD‐fed dams had significantly higher weight, lean mass, fat mass, and total body water mass compared to CD‐fed dams. They also had higher fat mass percentage and lower lean mass percentage. **p < 0.01, ***p < 0.001, ****p < 0.0001. Data were analyzed using t‐tests comparing LFD to CD and HFD to CD.

To model maternal undernutrition and overnutrition, dams were maintained on LFD, control‐fat (CD), or HFD throughout gestation and lactation. After 3 weeks on diet, HFD‐fed dams exhibited significantly increased body weight and altered body composition, including elevated fat mass and reduced lean mass percentage, relative to CD‐fed controls (Figure 1A,B). In contrast, LFD‐fed dams did not differ significantly in weight or body composition compared to CD‐fed dams.

**FIGURE 2:**
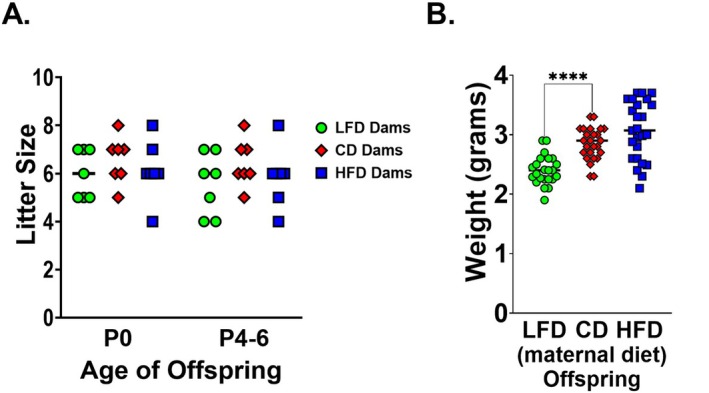
LFD‐fed dams had significantly smaller litter sizes and lighter offspring at time of sepsis. After 3 weeks of dietary intervention, C57BL/6 dams were bred with C57BL/6 males. Dams remained on their prescribed diet throughout gestation and lactation. Males were exposed only during the time of breeding and otherwise received standard chow. Offspring were counted and weighed at time of sepsis (P4–P6). (A) LFD‐fed dams had significantly smaller litter sizes compared to CD‐fed dams. There was no difference in HFD‐fed and CD‐fed dam litter sizes (*n* = 9–10 litters/group). (B) Offspring born to dams fed an LFD‐weighed significantly less than offspring born to dams fed a CD. Although it is not statistically significant, offspring of HFD‐fed dams trend toward weighing more than offspring of CD‐fed dams (*n* = 23–25/group). Bars represent median. Individual data points presented. *p < 0.05, **p < 0.01, ***p < 0.001, ****p < 0.0001. Data were analyzed using *t*‐tests comparing LFD to CD and HFD to control‐fat diet.

There was no significant difference in litter size on P0 and survival to P4–P6 (day of sepsis) (Figure 2A). Despite comparable maternal body composition, LFD‐fed dams produced significantly smaller offspring at the time of sepsis induction compared to CD‐fed dams (Figure 2B). HFD‐fed dams did not significantly differ in litter or offspring size compared to the other groups. Despite the lack of statistical significance, HFD‐fed dams trended toward heavier offspring. These data suggest that maternal diet macronutrient composition, rather than caloric intake, may underlie the observed offspring phenotype.

### 3.2 Maternal LFD and HFD Decreases Neonatal Survival During *S. epidermidis* Sepsis Independent of Bacterial Burden

**FIGURE 3:**
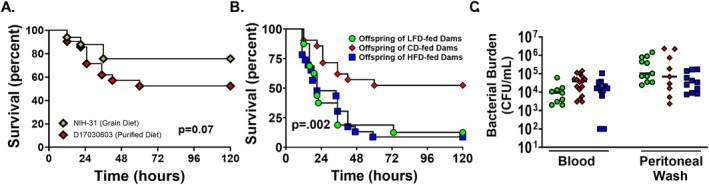
Offspring born to LFD‐ and HFD‐fed dams have significantly lower survival with no difference in bacterial burden. Sepsis was induced in P4‐P6 offspring with i.p. *Staphylococcus* *epidermidis* . Offspring were monitored for 5 days for survival. (A) Survival was not different in offspring of mice born to dams fed the standard grain diet (NIH‐31) or the purified control diet (D17030603) (*n* = 21–33/group). (B) Survival was significantly lower in offspring of LFD fed dams (13%) and HFD fed dams (9%) vs. offspring of CD fed dams (52%) (*n* = 16–23/group). **p < 0.01 using the log‐rank test. Results are combined from 3 experiments. (C) There was no difference in bacterial count in blood or peritoneal fluid (*n* = 12–24/group). Results are combined from 2 experiments.

To assess the impact of maternal diet on neonatal susceptibility to infection, offspring were challenged with intraperitoneal *S. epidermidis* and monitored for survival. First, we compared survival of offspring of dams fed the standard chow to offspring of dams fed the CD, and there was no significant difference in survival (Figure 3A). Offspring of both LFD‐ and HFD‐fed dams exhibited significantly reduced survival compared to offspring of CD‐fed dams (Figure 3B). The greatest decrease in survival is seen between 12‐ and 24‐h postinfection. These findings suggest that both maternal under‐ and overnutrition compromise neonatal host defense mechanisms.

To determine whether decreased survival was attributable to impaired bacterial clearance, bacterial load was quantified in blood and peritoneal fluid 12 h after *S. epidermidis* injection. No significant differences in colony‐forming units were observed across dietary groups in blood or peritoneal fluid (Figure 3C).

### 3.3 Maternal Dietary Fat Content and *S. epidermidis sepsis* Modulate Cytokine Profiles in Neonatal Serum

**FIGURE 4:**
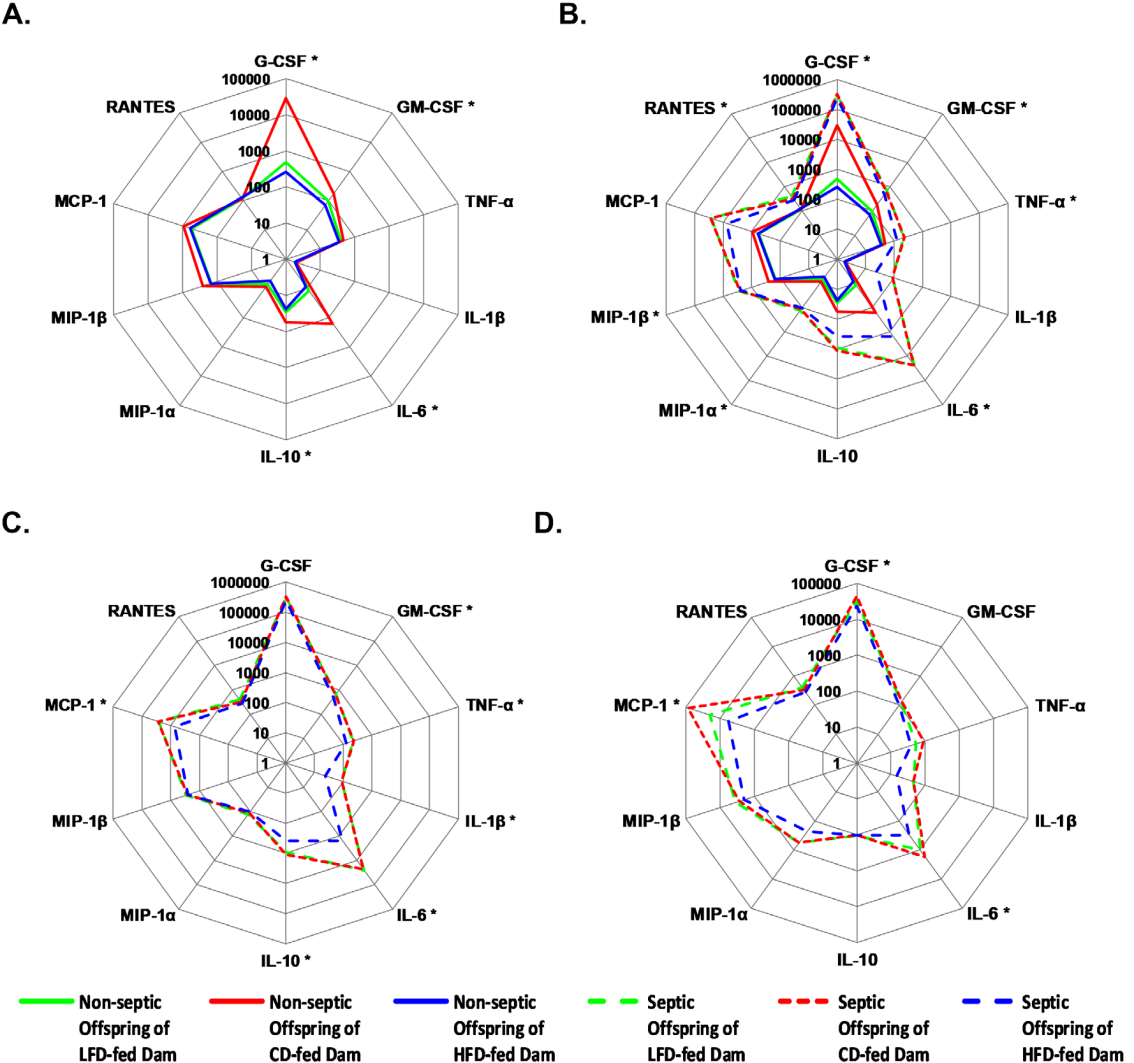
Maternal diet and *Staphylococcus epidermidis sepsis* influence serum cytokine levels. (A) In the absence of infection, offspring born to dams fed an LFD and HFD have significantly lower levels of G‐CSF, GM‐CSF, IL‐6, and IL‐10 compared to offspring born to dams fed a CD (*n* = 8/group; offspring from one to two litters). Data were analyzed using *t*‐tests to compare non‐septic offspring of LFD dams or HFD dams to offspring of CD dams. (B) Offspring of LFD, CD, and HFD‐fed dams have significantly higher levels of serum cytokines 12 h after *S. epidermidis* sepsis (*n* = 8/group; offspring from two litters). Data were analyzed using Two‐way ANOVA to compare nonseptic and septic offspring of LFD dams or HFD dams to offspring of CD. (C) Septic offspring of LFD fed dams do not have significantly different serum cytokine levels compared to septic offspring of CD fed dams at 12 h after sepsis. Septic offspring of HFD fed dams have significantly lower levels of key cytokines that regulate innate immune response compared to septic offspring of CD fed dams at 12 h after sepsis. (D) Septic offspring of LFD‐fed dams do not have significantly different peritoneal wash cytokine levels compared to septic offspring of CD‐fed dams at 12 h after sepsis. Septic offspring of HFD‐fed dams have significantly lower levels of G‐CSF, IL‐6, and MCP‐1 compared to septic offspring of CD fed dams at 12 h after sepsis. Data were analyzed using one‐way ANOVA to compare nonseptic and septic offspring of LFD dams or HFD dams to offspring of CD dams. Data are presented on a radar chart. Solid lines represent median of non‐septic mice and dashed lines represent median of septic mice. *p < 0.05, green lines = offspring of LFD dams; red lines = offspring of CD dams; blue lines = offspring of HFD dams.

We hypothesized that maternal malnutrition causes a dampened immune response in neonates. In the absence of infection, offspring of LFD‐ and HFD‐fed dams exhibited significantly reduced serum levels of key cytokines involved in innate immunity, including G‐CSF, GM‐CSF, IL‐6, and IL‐10, compared to CD‐fed dam offspring (Figure 4A). Following infection, all neonatal groups mounted significant increases in serum cytokines and chemokines, including G‐CSF, GM‐CSF, TNF‐α, IL‐6, MIP‐1α, MIP‐1β, and RANTES (Figure 4B). This confirms the activation of a systemic inflammatory response to *S. epidermidis* across maternal dietary conditions.

Despite similar bacterial loads, septic offspring of HFD‐fed dams exhibited blunted cytokine responses compared to CD‐fed dams. At 12 h after *S. epidermidis* infection, offspring of HFD‐fed dams had significantly reduced levels of serum GM‐CSF, TNF‐α, IL‐1β, IL‐6, IL‐10, and MCP‐1 compared to offspring of CD‐fed dams (Figure 4C). Similarly, septic offspring of HFD‐fed dams had significantly reduced levels of peritoneal wash G‐CSF, IL‐6, and MCP‐1 compared to septic offspring of CD‐fed dams (Figure 4D). These data suggest that maternal overnutrition impairs neonatal cytokine signaling during infection, potentially contributing to decreased survival.

In contrast, septic offspring of LFD‐fed dams demonstrated cytokine and chemokine profiles comparable to offspring of CD‐fed dams at 12 h after *S. epidermidis* infection. Septic offspring had robust elevations in IL‐1β, IL‐6, IL‐10, and MCP‐1 (Figure 4C). There was no difference in peritoneal wash cytokine levels in septic offspring of LFD and CD‐fed dams (Figure 4D). These findings indicate that while maternal LFD alters several basal serum cytokine levels, it does not significantly impair the acute cytokine response to infection.

### 3.4 Maternal Dietary Fat Content Reprograms the Neonatal Serum Metabolome

**FIGURE 5:**
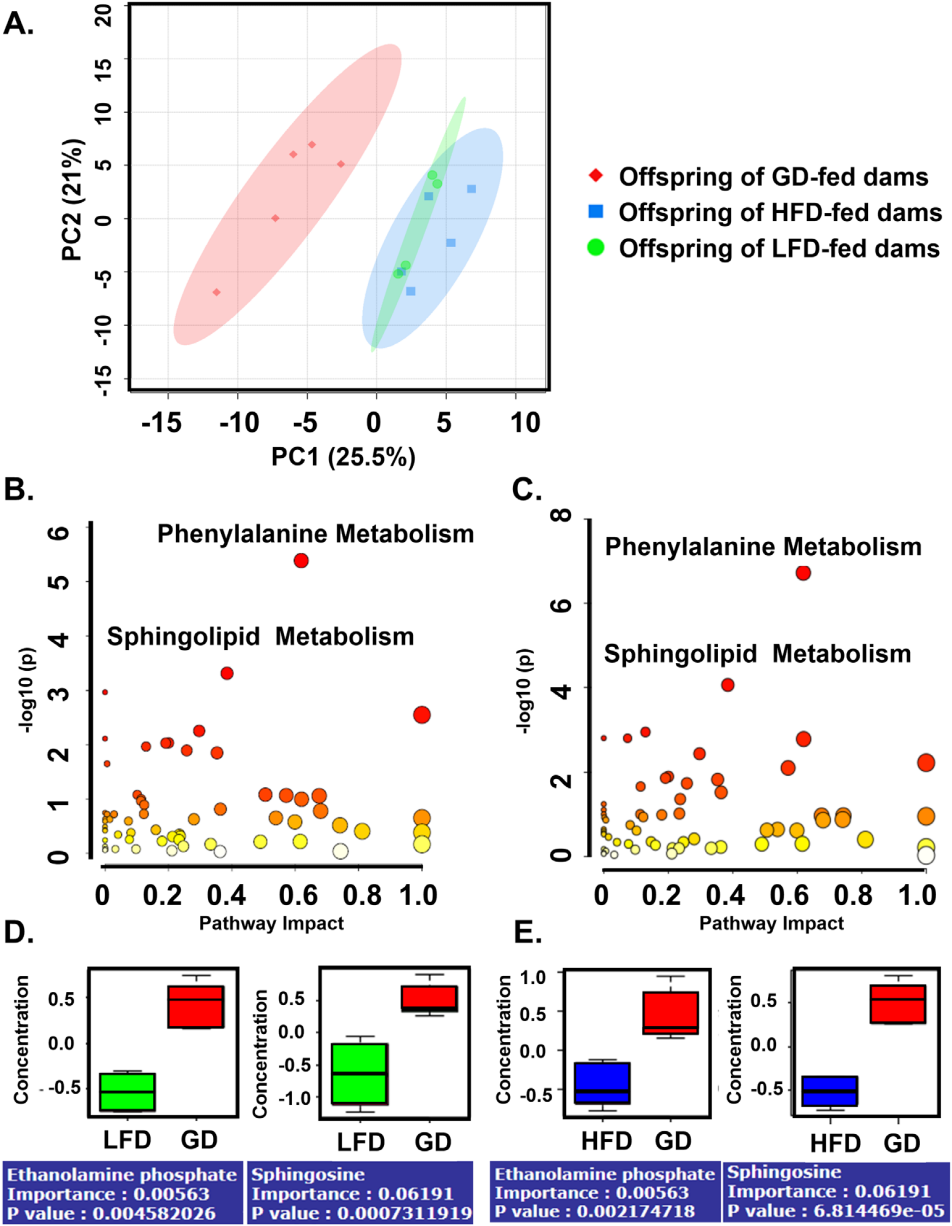
Offspring born to dams fed a LFD or HFD have significantly different serum metabolomes compared to offspring born to dams fed a GD. Serum from non‐septic P4–P6 offspring was pooled (3–6 offspring per sample) and analyzed using UHPLC–MS/MS performed by Metabolon Inc. (A) Unsupervised principal‐component analysis (PCA) demonstrates a significant difference in serum metabolome in offspring of LFD‐, GD‐, and HFD‐fed dams. (B) Pathway impact analysis of offspring of LFD‐fed dams. (C) Pathway impact analysis of offspring of HFD‐fed dams. (D) Sphingolipid metabolites are lower in offspring of LFD‐fed dams. (E) Sphingolipid metabolites are lower in offspring of HFD‐fed dams.

Given the lack of a clear mechanism for decreased survival in septic pups born to dams fed a LFD or HFD, we sought to determine if changes in the neonatal metabolome could be playing a role. Untargeted metabolomics was performed on serum from offspring without sepsis to evaluate for baseline differences. An unsupervised principal‐component analysis revealed there was a significant difference in the composition of the serum metabolome in offspring born to GD‐fed dams, LFD‐fed dams, and HFD‐fed dams (Figure 5A, *p* = 0.004): [PERMANOVA] *F*‐value: 5.8184; *R* ^2^: 0.51406; *p*‐value (based on 999 permutations): 0.004. The serum metabolome of offspring born to GD‐fed dams is significantly different than offspring of LFD‐fed dams: [Pairwise PERMANOVA] *F*‐value: 5.8339; *R* ^2^: 0.45457; *p*‐value (based on 999 permutations): 0.006. There was also a significant difference in the composition of the serum metabolome in offspring born to GD‐fed dams and HFD‐fed dams (Figure 5B, *p* = 0.012): [Pairwise PERMANOVA] *F*‐value: 9.38960; *R* ^2^: 0.53995; *p*‐value (based on 999 permutations): 0.009. Pathway enrichment analysis identified phenylalanine and sphingolipid metabolism as significantly impacted in both malnourished groups, suggesting shared metabolic disruptions that may underlie altered immune phenotypes (Figure 5C,D). There were multiple metabolites in the sphingolipid metabolism pathway that were altered in the same direction in offspring of LFD and HFD‐fed dams (Figure 5E,F).

### 3.5 Maternal Dietary Fat Content Does Not Impact Diversity of the Offspring Colonic Microbiome

**FIGURE 6:**
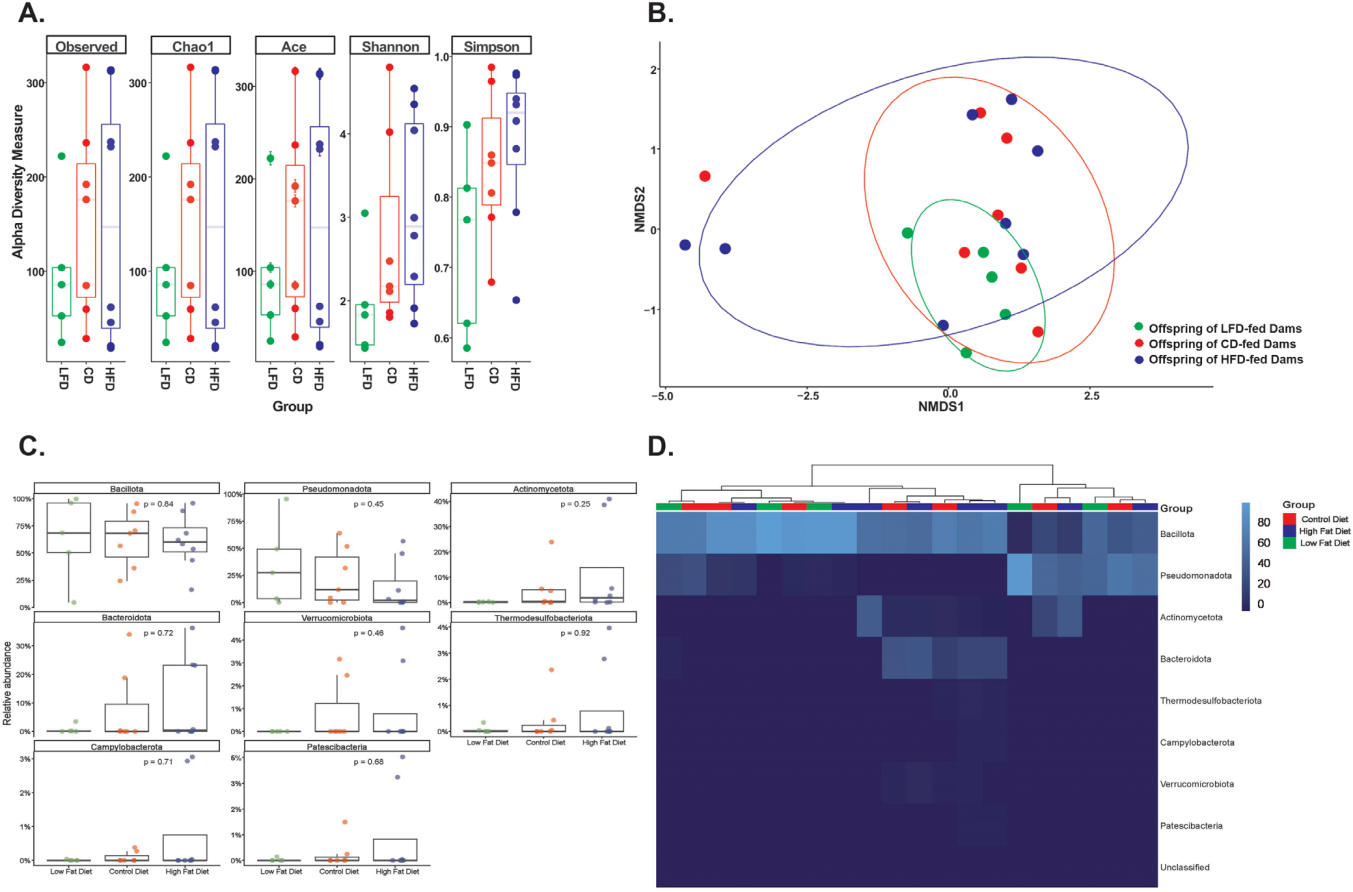
Maternal dietary fat content does not significantly impact colonic microbiome composition in nonseptic P4–P6 offspring (*n* = 5–8 per group). (A) Multiple measures of α‐diversity (Observed, Chao1, Ace, Shannon, and Simpson) demonstrate no significant difference in among offspring of LFD, CD, and HFD‐fed dams. Observed *p* = 0.72 by Kruskal–Wallis, Chao1 *p* = 0.72 by Kruskal–Wallis, Ace *p* = 0.72 by Kruskal–Wallis, Shannon *p* = 0.15 by Kruskal–Wallis, Simpson *p* = 0.22 by one‐way ANOVA. (B) Nonmetric multidimensional scaling (NMDS) plot demonstrates no significant difference in β‐diversity among offspring of LFD, CD, and HFD‐fed dams. *p* = 0.37 by PERMANOVA. (C) Phyla relative abundance is not significantly different among offspring of LFD, CD, and HFD‐fed dams by Kruskal–Wallis. (D) Heatmap visualization reveals an absence of phylum‐level clustering across offspring from dams fed LFD, CD, or HFD diets.

As there was a significant difference in offspring serum metabolome, we performed 16S rRNA sequencing on whole colons excised from neonates without infection to determine whether there was a difference in their colonic microbiome that could contribute to serum metabolomic differences. There were no differences in overall colonic microbiome composition between the GD‐fed and CD‐fed offspring (Figure S1), so we included samples from CD‐fed offspring in our subsequent colonic microbiome analysis to most closely match the dietary content between groups. There was no statistical difference in α‐diversity within colon samples of offspring of LFD‐fed, CD‐fed, and HFD‐fed dams on any of the indices of α‐diversity we analyzed (Figure 6A). Nonmetric multidimensional scaling (NMDS) plot with statistical analysis revealed there was also no difference in β‐diversity between colon samples from offspring of LFD, CD, and HFD‐fed dams (Figure 6B). Colon samples from offspring of LFD‐, CD‐, and HFD‐fed dams showed comparable phylum‐level relative abundances (Figure 6C), and no phylum‐level clustering was evident within dietary groups (Figure 6D).

## 4 Discussion

This study demonstrates that both maternal undernutrition and overnutrition, modeled through LFD and HFD respectively, significantly impair neonatal immune responses and increase susceptibility to sepsis. Despite divergent effects on maternal body composition, both maternal LFD and HFD resulted in decreased neonatal survival following *S. epidermidis* infection, independent of bacterial burden. These findings suggest that maternal diet exerts a profound influence on neonatal immune programming, likely through mechanisms beyond simple nutrient availability or pathogen load.

This study is the first, to our knowledge, to examine the impact of maternal dietary fat composition on neonatal sepsis outcomes. Maternal malnutrition—defined as an imbalance in calories, macronutrients, vitamins, and minerals during gestation and lactation—can arise from both low‐fat, grain‐based diets common in resource‐limited settings and high‐fat, ultra‐processed diets. Adequate nutrition is essential for fetal growth, immune development, and postnatal survival [29, 30, 31, 32]. For our control diet, we used a custom purified diet that matched the percentage of kcal from fat in the standard chow provided at our institution. There was no statistical difference in septic offspring survival between these two maternal diets; however, survival trended lower using the CD. This supports our use of the custom purified 18% kcal from fat diet as our control diet. Interestingly, the 10% kcal from fat diet is used as a control for the 60% kcal from fat diet by other researchers. Similar to our findings, two studies reported no difference in litter size when dams are fed a 10% or 60% kcal from fat diet [33, 34]. Chen et al. found no difference in P2 offspring born to dams fed a 10% or 60% kcal from fat diet [33]. This difference from our findings, in which offspring of dams fed LFD (10% kcal from fat) weighed significantly less, may be explained by the day of life in which the offspring were weighed. It is possible that the difference in weights we observed is due to differences in milk composition between LFD and CD‐fed dams. Despite no differences in maternal weight or body composition between LFD and CD groups, offspring from LFD‐fed dams had reduced size and survival at P4–P6. It may be that the 10% kcal from fat diet is insufficient to support gestation and lactation. Alternatively, we found that HFD resulted in increased weight and altered body mass composition in dams, with a strong trend toward heavier offspring. This is consistent with some studies but contradicts others, which found lower offspring weight [35]. These variations in both diets may be due to differences in genetic background of mice, time spent on the diet, or variation in diet composition between vendors. Ultimately, offspring of dams fed a LFD or HFD had significantly lower survival from sepsis suggesting that maternal diet fat composition plays an important role in neonatal susceptibility to infection.

Lai et al. found a relatively sublethal dose of 3.5 × 10^7^ cfu/mL of *S. epidermidis* i.p. produced bacteremia with an inflammatory response in P4 C57BL/6 neonatal mice [36]. They found that serum bacterial counts were significantly elevated at 2 h postinfection with a mild increase above the detection threshold at 14 h and below the detection level by 48 h following infection. It is possible that we did not find any differences in serum bacterial cfus in septic offspring because of the timepoint we used. Future studies can consider earlier timepoints as later timepoints will be confounded by survival bias as animal deaths start to occur around 12 h after infection.

Offspring of both LFD‐ and HFD‐fed dams exhibited reduced basal levels of key cytokines, including IL‐6, IL‐10, G‐CSF, and GM‐CSF, indicating a dampened innate immune system before infection. Manches et al. did not find any difference in cord blood cytokines among healthy neonates in relation to maternal pre‐pregnancy BMI, third‐trimester maternal weight, or birth weight [37]. Serum IL‐6 is correlated with infection, specifically chorioamnionitis, but not maternal diabetes [38]. Serum IL‐10 has been found to be decreased in the serum of children with obesity and rats fed a HFD [39]. Tissue‐specific IL‐10 levels are also reduced in neonatal mice born to dams fed a HFD [40]. IL‐10 is an anti‐inflammatory cytokine that helps modulate the inflammatory response. G‐CSF and GM‐CSF are involved in hematopoiesis, specifically the production and maturation of neutrophils and macrophages. Taken together, this dysregulated basal immune phenotype may predispose neonates to inadequate responses during early infection, which could contribute to the observed decrease in survival.

Neonatal mice, regardless of maternal diet, had significant elevation of a wide array of serum cytokines and chemokines 12 h following *S. epidermidis* sepsis. Consistent with these findings, Lai et al. found most cytokines and chemokines were significantly increased following *S. epidermidis* sepsis. Nearly all cytokines and chemokines peaked around 14 h postinfection, except for IL‐6, which peaked at 2 h postinfection, and they returned to baseline by 48 h. Interestingly, while both LFD and HFD offspring demonstrated decreased survival, their cytokine responses to sepsis diverged. Offspring of HFD‐fed dams exhibited significantly blunted cytokine and chemokine responses during infection, including reduced levels of serum GM‐CSF, TNF‐α, IL‐1β, IL‐6, IL‐10, and MCP‐1, and peritoneal G‐CSF, IL‐6, and MCP‐1. These findings are consistent with prior studies demonstrating impaired monocyte and dendritic cell responses in neonates born to obese mothers and suggest that maternal overnutrition may induce a state of immune tolerance or exhaustion in the offspring [22, 41]. In contrast, LFD offspring mounted cytokine responses comparable to controls during sepsis. Maternal diet clearly impacted offspring cytokine levels, but there was not a clear relationship between these alterations and sepsis survival, suggesting other mechanisms were involved.

Metabolomic profiling revealed that maternal diet significantly altered the neonatal serum metabolome, with shared significant pathways including phenylalanine and sphingolipid metabolism. These pathways are known to influence immune cell signaling, membrane dynamics, and inflammatory responses, and may represent mechanistic links between maternal diet and offspring immune function [42, 43, 44]. Sphingolipid metabolism, specifically the shingosine‐1‐phosphate metabolite, has been indicated as a potential biomarker and therapeutic target for patients with sepsis [45, 46, 47]. The shared metabolic disruptions observed in both LFD and HFD offspring suggest that distinct nutritional insults may converge on common immunometabolic pathways. Future studies will include targeted metabolomic studies to further explore these pathway differences at a systemic, tissue, and cellular level.

Several studies have described the impact of maternal diet on infant gut microbiome composition [48, 49, 50]. A nonhuman primate study demonstrated that maternal HFD during gestation has a long‐standing impact that cannot be fully reversed with postnatal weaning to a CD [51]. Human studies have demonstrated meconium microbiome differences in neonates born to mothers that reported a HFD during pregnancy [50]. Babu et al. found maternal HFD altered offspring colonic microbiome by 1 week of age [52]. Our study did not find a difference in the colonic microbiome of neonatal mice based on maternal dietary fat content or between offspring born to dams on grain‐based or purified control fat diets. This is surprising, as grain diets use animal and plant by‐products with variable ingredients as a source of soluble and insoluble fiber while purified diets use insoluble cellulose as the primary fiber source. A previous study in adult male mice found significant differences in colonic microbial communities between mice fed grain‐based and purified diets [53]. However, microbial communities are known to have low overall diversity until P14, when pups start coprophagy and have a diet that contains more solid food [54]. Therefore, it is possible that the colonic microbial communities found at this early timepoint (P4–P6) are more reflective of nondietary factors, including institution‐ or cage‐specific effects. It is also possible that any diet‐driven changes fell below the limit of detection of 16S sequencing.

Maternal diet is also known to impact the components of breastmilk [55, 56, 57]. Maternal breastmilk impacts the composition of the infant gut microbiome and metabolome and reduces the risk of sepsis in preterm infants [58, 59, 60]. Our study provides evidence that maternal diet impacts outcomes during neonatal sepsis, but it does not distinguish between the impact of gestational maternal diet and postnatal maternal diet. Maternal breastmilk composition may be a primary determinant of neonatal immune responses, metabolomic profiles, and gut–microbial communities, with the potential to influence sepsis outcomes. To distinguish the effects of in utero exposure from those of postnatal maternal diet and breastmilk composition, future studies will incorporate cross‐fostering experiments.

This study has several limitations. First, while we observed significant changes in cytokine levels and metabolite profiles, we did not assess cellular immune responses or tissue‐specific inflammation, which may provide further mechanistic insight. Second, our model focused on a single pathogen and time point; future studies should evaluate whether these findings generalize to other infectious challenges and developmental stages. Finally, while our dietary models reflect macronutrient extremes, they do not capture the full complexity of human malnutrition, including micronutrient deficiencies and dietary diversity.

## 5 Conclusion

Our findings highlight the critical role of maternal nutrition in shaping neonatal immune competence and susceptibility to infection. Offspring of dams fed a LFD or HFD had significantly lower survival from sepsis without a difference in bacterial burden. While offspring of LFD‐fed dams had a similar cytokine response as offspring of dams fed a CD, the offspring of HFD‐fed dams had lower cytokine levels during sepsis, consistent with a dampened immune response. These offspring also had alterations in shared metabolic pathways that may provide a mechanistic reason for their decreased survival during sepsis. These results underscore the importance of optimizing maternal diet during pregnancy and lactation, not only for fetal growth but also for the development of a robust and responsive immune system in the offspring.

## Author Contributions

**Lauren Bodilly:** conceptualization, data acquisition, and data analysis and interpretation. **Sarah Weiner:** data acquisition. **Jennifer Bermick:** conceptualization and data analysis and interpretation. All authors were involved in drafting the manuscript and revisions.

## Funding

This work was supported by the Eunice Kennedy Shriver National Institute of Child Health and Human Development (NICHD) (5 K12 HD027748‐32), National Institute of Allergy and Infectious Diseases (NIAID) (R01AI150687 to J.B.).

## Conflicts of Interest

The authors declare no conflicts of interest.

## Supporting information


**Figure S1:** Colonic microbiome composition in nonseptic P4–P6 offspring remains largely unchanged between maternal purified and grain diets (*n* = 6–7 per group).

## Data Availability

The data that support the findings of this study are available in the Materials and Methods, Results, and/or Supporting Information of this article.
