# Supplementary figures and images for: Maternal Low‐Fat and High‐Fat Diet Decreases Survival and Alters Cytokine Signaling in Neonatal Mice With Staphylococcus epidermidis Sepsis
Source: FASEB J. 2026 Apr 13;40(8):e71794. doi: 10.1096/fj.202502656RR (PMC13071550; doi:10.1096/fj.202502656RR)

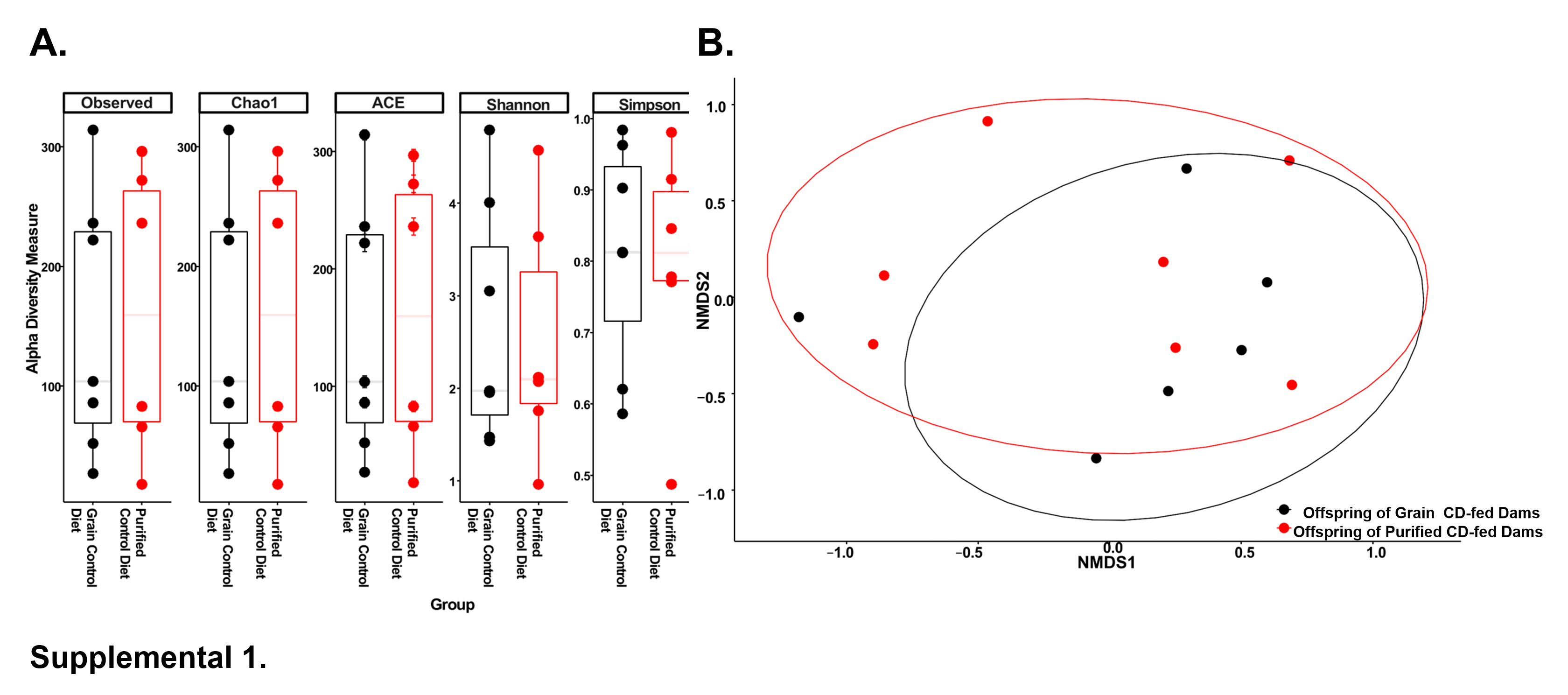

Supplement: Supplementary file 1 — Figure S1: Colonic microbiome composition in nonseptic P4–P6 offspring remains largely unchanged between maternal purified and grain diets (n = 6–7 per group). [file FSB2-40-e71794-s001.tif]
